# Molecular mechanisms underlying the potential anticancer activity of *Pulicaria crispa* hexane fraction in HCT116 cancer cells

**DOI:** 10.1007/s13205-025-04423-1

**Published:** 2025-07-15

**Authors:** Hamies B. Nabil, Emad Elzayat, Fatma Abo-Elghiet, Nourhan Hassan

**Affiliations:** 1 Medical Science Department, Faculty of Dentistry, The British University in Egypt, Cairo, Egypt; 2 Biotechnology Department, Faculty of Science, Cairo University, Giza, Egypt; 3 Department of Pharmacognosy and Medicinal Plants, Faculty of Pharmacy (Girls), Al-Azhar University, Cairo, Egypt

**Keywords:** *Pulicaria crispa*, Cancer, HCT116, Cytotoxicity, Apoptosis

## Abstract

Given the high mortality rate associated with tumors and the severe side effects of current treatments, scientists are exploring alternative therapies with fewer adverse effects. They are increasingly turning to natural remedies, much like our ancestors who used plant extracts to treat various ailments long before understanding the underlying mechanisms. Even though they did not know exactly why these plants treated those diseases then, we have the privilege of testing these plants and discovering the active ingredients responsible for these effects. This study aims to investigate the anticancer mechanisms of *Pulicaria crispa* hexane fraction (Hex F) against human colorectal cancer cells and elucidate its molecular pathways of action. The methanol extract of *P. crispa* and its fractions were evaluated for cytotoxic activity using MTT assay against HepG2, HCT116, and Hep-2 cancer cell lines, with oral epithelial normal cells (OEC) as controls. The most potent fraction (Hex F) was further analyzed using flow cytometry for cell cycle and apoptosis analysis, qRT-PCR for gene expression profiling, ELISA for protein quantification, and biochemical assays for oxidative stress and glycolytic enzyme activities. Hex F demonstrated significant cytotoxicity against HCT116 cells with an IC_50_ of 39.4 μg/mL and a selectivity index of 1.76 indicating preferential toxicity toward cancer cells. Flow cytometry analysis revealed G_2_/M phase cell cycle arrest and significant induction of apoptosis. Gene expression analysis showed significant upregulation of pro-apoptotic genes p53, caspase-8, and caspase-9, while anti-apoptotic Bcl2 was downregulated). Protein analysis confirmed increased caspase-3 and caspase-7 activities, accompanied by enhanced anti-inflammatory response with increased IL-10 and decreased IL-4 levels. Oxidative stress markers indicated cellular damage with decreased GSH and SOD levels, while MDA increased significantly. Glycolytic enzyme activities were substantially reduced, with PK, Aldolase, and LDH activities decreased, suggesting metabolic disruption. GC–MS analysis identified β-sitosterol (17.89%), phytol (15.65%), stigmasterol (13.13%), and lupeol (12.89%) as major bioactive compounds. These findings demonstrate that *P. crispa* Hex F exerts anticancer effects through multiple mechanisms including cell cycle arrest, apoptosis induction, oxidative stress generation, and metabolic disruption, supporting its potential as a natural anticancer therapeutic agent.

## Introduction

Cancer remains one of the leading causes of death worldwide, with recent global cancer statistics from 2024 indicating over 20 million new cases annually and approximately 9.7 million cancer-related deaths (Abed et al. 2021; Siegel et al. 2020). Among the deadliest cancers are colorectal cancer, which includes malignancies in colon and rectum with a 5-year survival rate of only 65% when diagnosed at advanced stages (Park et al. 2014; Marley and Nan 2016), and laryngeal cancer, a prevalent form of oral cancer that presents substantial health challenges affecting over 180,000 individuals globally each year (Eskander et al. 2018; Igissin et al. 2023). Hepatocellular carcinoma, the most common form of liver cancer, also contributes significantly to global cancer mortality due to its high incidence and insidious progression with incidence rates increasing by 2–3% annually in developed countries (Llovet et al. 2021; Yang et al. 2019; Foglia et al. 2023). Traditional cancer treatments, such as chemotherapy and surgery, often result in severe side effects and limited effectiveness, prompting increased interest in natural products as potential therapeutic alternatives with recent studies showing that 70% of patients experience significant quality of life improvements when natural products are integrated into conventional treatment protocols (Llovet et al. 2021; Chehelgerdi et al. 2023; Zafar et al. 2025; Anand et al. 2023). Notably, approximately 60% of anticancer therapies are derived from natural products, including flavonoids, alkaloids, tannins, and terpenoids, which are known for their anticancer properties and reduced side effects with over 250 natural product-derived compounds currently in clinical trials for cancer treatment (Ali Abdalla et al. 2022; Sharifi-Rad et al. 2019).

Recent advances in natural product research have highlighted the importance of plant secondary metabolites in cancer therapy, with studies from 2023 to 2024 demonstrating that combination therapies incorporating natural compounds can enhance treatment efficacy while reducing systemic toxicity (Rao et al. 2025; Mardiana et al. 2025; Ghosh et al. 2025). Phytochemical investigations have revealed that terpenoids and sterols, particularly β-sitosterol and stigmasterol, exhibit potent anticancer activities through multiple molecular pathways including apoptosis induction, cell cycle arrest, and angiogenesis inhibition (Mahmood et al. 2024; Alvarez-Sala et al. 2019; Wang et al. 2023; Laka et al. 2022; Awad and Fink 2000). Contemporary research has also emphasized the role of oxidative stress modulation and metabolic reprogramming as key mechanisms underlying the anticancer effects of plant-derived compounds (Shukla et al. 2025; Dong et al. 2025).

*Pulicaria crispa* (*P. crispa*), commonly known as Gethgath, is a member of the Compositae (Asteraceae) family and is traditionally used in Egypt, Sudan, and Saudi Arabia for treating various ailments including inflammatory conditions, digestive disorders, and skin diseases (Kasote et al. 2024a; Dekinash et al. 2018). *P. crispa* is rich in secondary metabolites, such as terpenes (e.g., phytol), sterols (e.g., stigmasterol, β-sitosterol, and lupeol), alkaloids (e.g., pulicaridine and pulicarine), phenolic compounds (e.g., chlorogenic acid, caffeic acid, and gallic acid), and flavonoids (e.g., kaempferol, quercetin, and luteolin), with recent phytochemical studies identifying over 50 bioactive compounds in various *Pulicaria* species (Razgonova et al. 2021; Prommaban et al. 2020; Kasote et al. 2024b; AlZain et al. 2023; Liu et al. 2010). These metabolites exhibit various biological activities, including antibacterial, antifungal, antiviral, antioxidant, anticancer, and anti-inflammatory effects with recent studies demonstrating IC_50_ values ranging from 10 to 100 μg/mL against various cancer cell lines (AlZain et al. 2023; Evidente 2024; Bhattacharjya et al. 2025). 

Recent investigations into *Pulicaria* species have revealed promising anticancer potential, with *P. undulata* and *P. dysenterica* showing significant cytotoxic effects against breast, lung, and colon cancer cells through mechanisms involving DNA damage, mitochondrial dysfunction, and immune system modulation (Nasr 2024; Mohamed et al. 2025; Barabadi et al. 2025). Studies published in 2023 have demonstrated that *Pulicaria* extracts can sensitize cancer cells to conventional chemotherapy drugs, potentially allowing for dose reduction and decreased side effects (Talib et al. 2021; Barnawi and Ali 2019). Furthermore, recent research has highlighted the synergistic effects of multiple bioactive compounds in *Pulicaria* extracts, suggesting that whole plant extracts may be more effective than isolated compounds (Al-Maqtari et al. 2021; Vaou et al. 2022).

Given its historical use and bioactive compounds, *P. crispa* represents a promising candidate for anticancer drug discovery with recent computational studies predicting high binding affinities of its major compounds to key cancer-related protein targets (Chunarkar-Patil et al. 2024; Asma et al. 2022a). In this study, the methanol extract of *P. crispa*, along with its fractions of varying polarities, was tested against multiple cancer cell lines and evaluated for cytotoxic effects on normal cells. The most potent fraction was further analyzed to determine its secondary metabolite composition. Comprehensive analyses were then conducted, including cell cycle analysis, apoptosis induction, gene expression profiling, modulation of glycolytic pathway, oxidative stress evaluation, and measurement of inflammatory markers, to elucidate the underlying mechanisms of action.

## Materials and methods

### Plant material and extraction method

The methanol extract and its fractions from *P. crispa* used in this study were prepared as part of our previous work following standardized extraction protocols established by the World Health Organization for medicinal plant preparations (Abo-Elghiet et al. 2023; Larson et al. 2016). Building on that work, we utilized the same extract and the fractions for further biological investigations. Briefly, preparation involved plant collection, authentication, voucher specimen deposition, and extraction using 100% methanol according to the methodology described by Harborne (1984) for phytochemical extraction (Harborne 1984). Methanol extract (MeOH Ex) was then fractionated via liquid–liquid partitioning with hexane, dichloromethane, ethyl acetate, and water to yield four distinct fractions: hexane (Hex F), dichloromethane (DCM F), ethyl acetate (EtOAc F), and aqueous (H_2_O F) using the sequential fractionation protocol established by Kupchan and Tsou (1973) (Emran et al. 2015; Kupchan et al. 1973).

### Cell lines and cell culture

In this study, various cell lines were obtained from the NAWAH Research Centre, including hepatocellular carcinoma cell line (HepG2), human colorectal carcinoma cell line (HCT116), human laryngeal cancer cell line (Hep2), and oral epithelial normal cells (OEC). These cell lines were cultured in Dulbecco’s modified Eagle’s medium (DMEM high glucose from Gibco®), supplemented with 10% fetal bovine serum (FBS, Thermo Fisher Scientific®, USA) and 1% antibiotic/antimycotic mixture (Lonza®, USA) following the standard cell culture protocols as described by Freshney (2010) (Freshney 2010). The cultures were maintained in a humidified incubator set at 37°C with 5% CO_2_, ensuring optimal growth conditions. Sub-culturing was performed when the cells reached 80–90% confluence. Cells were washed with PBS (Capricorn Scientific®) and detached using Trypsin EDTA (Lonza®, USA). All procedures were conducted in a class II laminar flow hood equipped with a ULPA filter to maintain sterility according to biosafety guidelines established by the CDC and NIH (Jagtap et al. 2023).

### Cytotoxicity (MTT assay)

For this study, a 96-well flat-bottom micro-titer plate was used, with 1 × 10^4^ cells/well seeded and allowed to attach for 24 h following the standardized MTT protocol established by Mosmann (1983) and modified by Berridge et al. (2005) (Mansour et al. 2021; Berridge et al. 2005; Mosmann 1983; Ghasemi et al. 2021). Serial dilutions of *P. crispa* MeOH Ex and its different fractions were prepared at concentrations of 100, 50, 25, 12.5, 6.25, and 3.125 μg/mL. Each concentration was tested in triplicate for each cell line. After the initial 24-h attachment period, the media were removed from the wells, and the cells were treated with the respective extracts for 48 h as recommended by the National Cancer Institute guidelines for in vitro anticancer drug screening (AlZain et al. 2023; Abbott et al. 2023). Positive controls consisted of untreated cancer cells, while negative controls contained wells with only media and no cells. Following treatment, the extract was removed and replaced with 5 mg/mL MTT solution, which was freshly prepared according to the protocol described by Stockert et al. (2012). The plate was incubated for 4 h, after which the MTT solution was discarded. To dissolve the formazan crystals, 100 μL of DMSO was added to each well, followed by a 20-min incubation and gentle vortexing.

Absorbance was measured using a microplate reader (Thermo Scientific Multiscan GO®, USA) at a wavelength of 570 nm with a reference wavelength of 630 nm as described by van Meerloo et al. (2011) (Mansour et al. 2021; Meerloo et al. 2011). The percentage of cell viability for each concentration of the extract was calculated using the formula: Cell viability (%) = (Absorbance of treated cells / Absorbance of control cells) × 100 (Meerloo et al. 2011; Sebaugh 2011). IC_50_ (the concentration of extract required to inhibit 50% of cell growth) was determined using non-linear regression analysis performed with GraphPad Prism software version 8 employing the four-parameter logistic curve fitting method as described by Sebaugh (2011); Raafat et al. 2023). Additionally, selectivity index (SI) was calculated by comparing the IC_50_ values of the methanol extract and its fractions on cancerous versus normal cell lines according to the methodology established by Koch et al. (2005). This allowed us to identify the most promising samples and cancer cell lines for further analysis (Abed et al. 2021) that were used in further experiments.

### Cell cycle analysis, apoptosis, and necrosis using flow cytometry

The HCT116 cells were treated with IC_50_ concentration of Hex F (39.4 µg/mL). The Propidium Iodide (PI) Flow Cytometry Kit for Cell Cycle Analysis (ab139418, Abcam®, UK) was used to determine the cell cycle phases following the protocol established previously (Kim and Sederstrom 2015; Roukos et al. 2015). HCT116 cells were fixed with ethanol and stored at 4°C. They were then rehydrated in PBS and stained with PI and RNase for 30 min according to the standardized protocol described by Kim and Sederstrom (2015). The PI fluorescence intensity was collected using the FL2 channel of a flow cytometer with 488 nm laser excitation. Cell cycle phases were analyzed using ModFit LT software with debris and doublet discrimination as recommended by the International Society for Analytical Cytology (Cossarizza et al. 2017). To assess apoptosis and necrosis, Annexin V-FITC Apoptosis Detection Kit (Catalog #: K101-25, Biovision®, USA) was used following the methodology established by Koopman et al. (1994) (Lakshmanan and Batra 2013). HCT116 cells were re-suspended in a binding buffer, and Annexin V-FITC along with PI according to the manufacturer's protocol with incubation times optimized based on Crowley et al. (2016). The mixture was incubated in the dark for 5 min. The binding of Annexin V-FITC was analyzed by flow cytometry using a FITC signal detector (FL1), and PI staining was analyzed using the phycoerythrin emission signal detector (FL2) with an excitation wavelength of 488 nm and an emission wavelength of 530 nm. For all measurements, the BD FACS Calibur flow cytometer was used, and assays were performed in triplicate. Untreated HCT116 cells were included as controls for both techniques with gating strategies established according to the guidelines published by Perfetto et al. 2004.

### Gene expression by qRT-PCR

Total RNA was extracted from both treated and untreated (control) HCT116 cells using the Qiagen® RNA extraction kit (Qiagen®, USA) following the protocol described by Chomczynski and Sacchi (2006). Concentration and purity of the RNA were assessed using a Nanodrop spectrophotometer and verified by 1% gel electrophoresis with quality criteria established by Fleige and Pfaffl (2006): A_260_/A_280_ ratio between 1.8–2.2 and A_260_/A_230_ ratio > 1.8 (Vermeulen et al. 2011). For reverse transcription, 5 μg of RNA was used with the iScriptTM One-Step RT-PCR Kit with SYBR® Green (BioRad®, USA), serving as a template to measure the expression of specific genes: Bcl2, p53, caspase-9 (Casp9), caspase-8 (Casp8), CDK2, and TopBp1. This was performed using the Rotorgene RT-PCR system (Qiagen®, USA) according to the MIQE guidelines established by Bustin et al. (2009).

Specific primers for the target genes listed in Table 1 were verified using NCBI Primer Blast. The relative expression levels were normalized using GAPDH as the housekeeping gene and calculated using the 2^−ΔΔCt^ method as described by Livak and Schmittgen (2001). The PCR cycler parameters were set as follows: an initial denaturation step at 95°C for 10 min, followed by 45 cycles of 95°C for 15 s, 60°C for 30 s, and 72°C for 30 s. After the final cycle, the temperature was gradually increased from 72°C to 95°C to generate the melting curve following the protocol optimization guidelines described by Ririe et al. (1997) (Mansour et al. 2021).

**Table 1:** Primer sequences used for qRT-PCR analysis

Genes	Primers
Bcl2	F 5’- ATCGCCCTGTGGATGACTGAGT -3’ R 5'- GCCAGGAGAAATCAAACAGAGGC-3'
p53	F 5’- CCTCAGCATCTTATCCGAGTGG -3’ R 5'- TGGATGGTGGTACAGTCAGAGC -3'
Casp8	F 5’- AGAAGAGGGTCATCCTGGGAGA-3’ R 5'- TCAGGACTTCCTTCAAGGCTGC -3'
Casp9	F 5’- GTTTGAGGACCTTCGACCAGCT-3’ R 5'- CAACGTACCAGGAGCCACTCTT -3'
CDK2	F 5’- ATGGATGCCTCTGCTCTCACTG -3’ R 5'- CCCGATGAGAATGGCAGAAAGC -3'
TopBp1	F 5’- GGACAACCACTTCAGAAGGAGC -3’ R 5'- CCTGGAGTTTCCAAGGCTGCAA -3'
GAPDH	F 5’- GTCTCCTCTGACTTCAACAGCG -3’ R 5’- ACCACCCTGTTGCTGTAGCCAA-3’

### Determination of caspase proteins and anti-inflammatory markers using the ELISA technique

To measure the activity of caspase-3 and caspase-7 in both treated and untreated cells, specific ELISA kits were used for each following standardized protocols established by Nicholson et al. (1995) for caspase activity measurement (Nicholson et al. 1995). The ELISA Kit (Catalog #KHO1091, Invitrogen®, USA) was employed for Casp3, while for Casp7, caspase-7 (Human) ELISA Kit (Catalog #E4295, BioVision®, USA) was used. The assays were conducted according to the manufacturer's protocols. The absorbance readings were obtained using a ROBONIK P2000 ELISA READER.

On the other hand, inflammatory markers IL10 and IL4 were also measured in both treated and untreated HCT116 cells in triplicates using the kits (ab46034–IL-10 Human ELISA Kit, Abcam®, UK) and (RayBio® Human IL-4 ELISA Kit) respectively following the cytokine measurement protocols established by Bienvenu et al. (2000). Sample preparation and storage conditions followed the guidelines established by de Jager et al. (2009) for cytokine stability and measurement accuracy (Jager et al. 2009).

### Determination of oxidative stress markers

To assess oxidative stress markers and antioxidant activity in HCT116 cells, reduced glutathione (GSH), superoxide dismutase activity (SOD), malondialdehyde (MDA), and catalase (CAT) were measured in both treated and untreated cells following established protocols for oxidative stress assessment as described by Halliwell and Gutteridge (2015). For these measurements, the following kits were used: GSH levels were determined using the Glutathione Colorimetric Assay Kit (Biovision®, USA) based on the method established by Ellman (1959). MDA, a marker of lipid peroxidation, was measured using the ab118970 Lipid Peroxidation (MDA) Assay Kit (Abcam®, UK) following the thiobarbituric acid reactive substances (TBARS) method described by Ohkawa et al. (1979). SOD activity was assessed using the Enzyme-linked Immunosorbent Assay Kit for Superoxide Dismutase (SOD) (Cloud-clone corp®, USA) based on the methodology established by McCord and Fridovich (1969), and CAT activity was evaluated using the ab83464 Catalase Activity Assay Kit (Abcam®, UK) following the protocol described by Aebi (1984). All measurements were performed colorimetrically in triplicate using the ROBONIK P2000 ELISA READER (ROBONIK, India) with quality control measures as recommended by Miller et al. (1993).

### Determination of glycolytic pathway enzymes

To evaluate the activities of key glycolytic enzymes in HCT116 cells, lactate dehydrogenase (LDH), pyruvate kinase (PK), and aldolase were estimated in both treated and untreated cells following established protocols for glycolytic enzyme activity measurement as described by Bergmeyer (1984) (Pomeranz and Meloan 1994; Bergmeyer 1984). These measurements were performed in triplicate using the following kits: LDH activity was determined using the ab102526 Lactate Dehydrogenase (LDH) Assay Kit (Abcam®, UK) based on the method established by Wroblewski and LaDue (1955). PK activity was assessed with the ab83432 Pyruvate Kinase (PK) Assay Kit (Abcam®, UK) following the protocol described by Brevet, et. al. (1975), and aldolase activity was measured using the ab196994 Aldolase Activity Assay Kit (Abcam®, UK) based on the methodology established by Seibert and Tracy (2014). All enzyme activities were quantified using the ROBONIK P2000 ELISA READER (ROBONIK, India).

### GC–MS analysis of Hex F

The chemical composition of *P. crispa* Hex F was previously determined using GC–MS following standardized protocols for plant extract analysis (Abo-Elghiet et al. 2023; Elsevier. 2011). Briefly, the GC–MS analysis was performed on an Agilent Technologies system equipped with a 7890B GC and a 5977A MS detector. A diluted sample was injected into an HP-5MS column and analyzed using a temperature gradient according to the methodology established by Adams (2007) for essential oil and plant extract analysis (Sparkman 1997). The identified compounds were compared to the Wiley and NIST Mass Spectral Library with identification criteria based on mass spectral matching scores > 80% and retention index comparison as described by Babushok et al. (2011).

#### Statistical analysis

The results from all experiments were expressed as mean ± standard deviation (SD) for all replicates. Statistical significance was determined using an independent t test performed with SPSS software version 28.0 following the guidelines established by Field (2013) for statistical analysis in biological research (Field 2012). A *p* value of less than 0.05 was considered statistically significant with effect sizes calculated using Cohen's d as described by Cohen (1988) (Cohen 2013). For multiple comparisons, Bonferroni correction was applied to control for Type I error as recommended by Dunn (1961).

## Results

### Cytotoxicity and selectivity index of *P. crispa* MeOH Ex and its fractions

The cytotoxic effects of *P. crispa* MeOH Ex and its fractions were evaluated using the MTT assay against three cancer cell lines, with a normal cell line as a control. The half-maximal inhibitory concentration (IC_50_), which measures the concentration needed to inhibit 50% of cell viability, and the selectivity index (SI), defined as the ratio of IC_50_ values for normal cells to cancer cells, were calculated to evaluate potency and selectivity (Table 2). Both Hex F and DCM F exhibited the most favorable IC_50_ values, indicating strong cytotoxic activity, and notable SI values, reflecting their selective toxicity toward cancer cells. Notably, Hex F demonstrated significant cytotoxicity against the HCT116 cell line with minimal toxicity on normal cells, indicating its potential for further development as a targeted anticancer agent.

**Table 2:** IC_50_ values and selectivity indices of *P. crispa* extracts and fractions against cancer and normal cell lines

Fraction	OEC IC_50_ (µg/mL)	Hep2 IC_50_ (µg/mL)	SI Hep2	HCT116 IC_50_ (µg/mL)	SI HCT116	HepG2 IC_50_ (µg/mL)	SI HepG2
MeOH Ex	1607	306.6	5.2	257.8	6.2	955.9	1.7
Hex F	68.89	74.07	0.9	39.4	1.7	41.32	1.6
DCM F	18.74	41.82	0.4	21.51	0.9	22.25	0.8
EtOAc F	870.2	557.3	1.6	999	0.9	766.3	1.1
H_2_O F	306.8	2647	0.1	58.12	5.3	1704	0.2

SI = Selectivity Index calculated as IC_50_ (normal cells)/IC_50_ (cancer cells). Methanol extract (MeOH Ex), hexane (Hex F), dichloromethane (DCM F), ethyl acetate (EtOAc F), and aqueous (H2O F)

### Effect of Hex F on cell cycle analysis, apoptosis, and necrosis

**Fig. 1:**
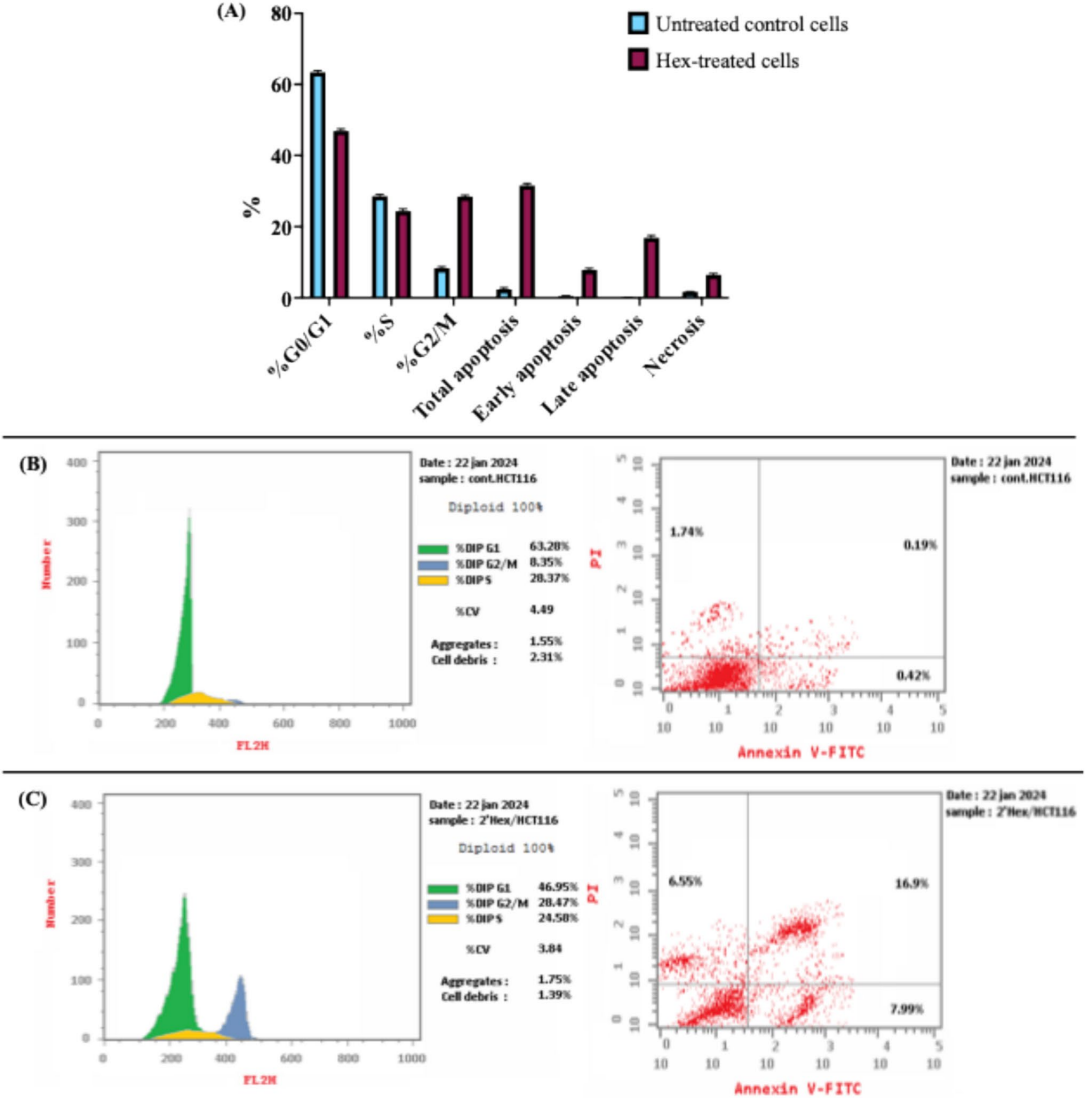
Flow cytometric analysis of cell cycle distribution and apoptosis in HCT116 cells treated with *P. crispa* Hex F. (A-C) HCT116 cells were treated with 39.4 µg/mL Hex F. **A** Percentage of cell populations in each cell cycle phase, and viable, early apoptotic, late apoptotic cells, and necrotic cells. Data are presented as the means ± SD of triplicate experiments. *p < 0.05, **p < 0.005, and ***p < 0.001 vs. control. **(B, C)**. **Left panel:** Typical cell cycle profiles. **Right panel:** representative scatter plots of PI (y-axis) vs. annexin V (x-axis). Quadrants represent: Q1 (necrotic cells, PI + /Annexin V-), Q2 (late apoptotic cells, PI + /Annexin V +), Q3 (viable cells, PI-/Annexin V-), Q4 (early apoptotic cells, PI-/Annexin V +). **B** Control untreated cells and **C** Cells treated with 39.4 μg/mL Hex F for 48 h

As shown in Table 3 and Fig. 1, the treatment of HCT116 cells with 39.4 µg/mL Hex F resulted in significant alterations in cell cycle dynamics. A statistically significant decrease in the percentage of cells in the G_0_/G_1_ and S phases was observed, accompanied by a corresponding increase in the G2/M phase compared to untreated control cells, indicating cell cycle arrest at the G_2_/M checkpoint with G_2_/M phase cells increasing from 8.3267 in control to 28.4433 in treated cells (*p* < 0.001).

**Table 3:** Cell cycle distribution and apoptosis analysis of HCT116 cells treated with *P. crispa* Hex F

	%G_0_/G_1_	%S	%G_2_/M	Total apoptosis	Early apoptosis	Late apoptosis	Necrosis
Untreated control cells	63.4233	28.57	8.3267	2.4300	0.5267	0.1833	1.7233
Hex F-treated cells	46.9467	24.4300	28.4433	31.5933	7.8300	16.8833	6.4400

Values represent the mean of three independent experiments. Statistical significance was determined using an independent t test

In parallel, apoptosis analysis revealed significant increases in both early and late apoptotic stages, as well as necrosis, compared to untreated control cells (Table 3 and Fig. 1). Early apoptotic cells increased from 0.5267 in control to 7.8300 in treated cells (*p* < 0.001), while late apoptotic cells increased from 0.1833 to 16.8833 (*p* < 0.001). Necrotic cells also showed a significant increase from 1.7233 to 6.4400 (*p* < 0.01).

### Effect of Hex F on gene expression of pro- and anti-apoptotic genes

**Fig. 2:**
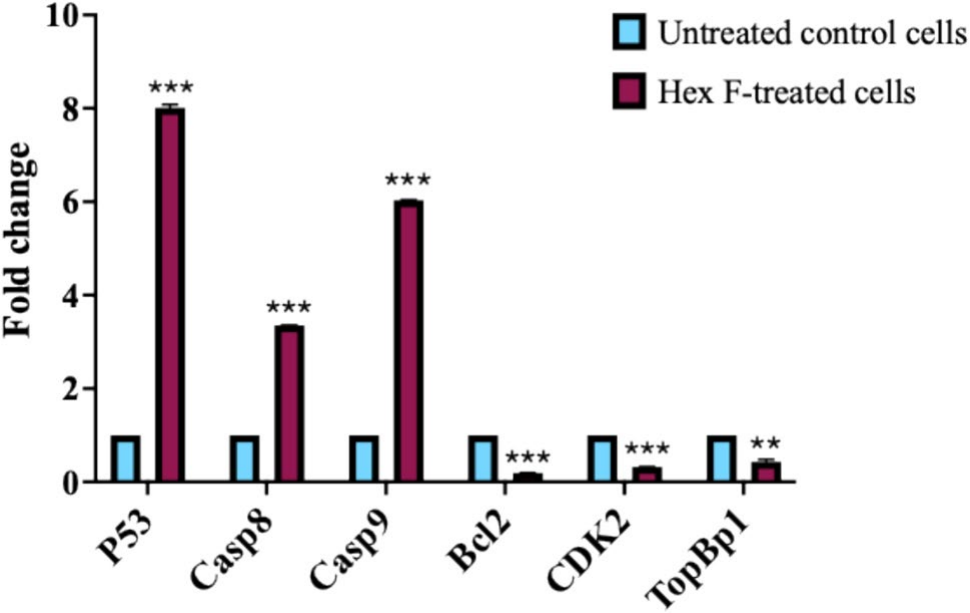
Gene expression analysis of apoptosis-related and cell cycle regulatory genes in HCT116 cells treated with *P. crispa* Hex F. Relative mRNA expression levels were determined by qRT-PCR using the 2^(-ΔΔCt) method with GAPDH as the housekeeping gene. Cells were treated with 39.4 μg/mL Hex F for 48 h. Pro-apoptotic genes showing significant upregulation: p53 (tumor suppressor gene), caspase-8 (initiator caspase for extrinsic apoptosis pathway), and caspase-9 (initiator caspase for intrinsic apoptosis pathway). Anti-apoptotic gene Bcl2 shows significant downregulation, cell cycle regulatory genes showing downregulation: CDK2 (cyclin-dependent kinase 2, essential for S-phase progression) and TopBP1 (DNA topoisomerase II-binding protein 1, involved in DNA replication and repair). Data represent mean ± SD of three independent experiments performed in triplicate. Statistical significance was determined using an independent t test: **p < 0.005, and ***p < 0.001 compared to untreated control. Error bars represent standard deviation

The qRT-PCR analysis of HCT116 cells treated with the IC_50_ of *P. crispa* Hex F demonstrated significant upregulation of pro-apoptotic genes (p53, Casp8, Casp9) and downregulation of the anti-apoptotic gene Bcl2, indicating apoptosis induction (Fig. 2). Furthermore, CDK2 and TopBP1 were significantly downregulated in treated cells compared to untreated controls, as illustrated in Fig. 2.

### Effects of Hex F on the activities of caspases and inflammatory markers

**Fig. 3:**
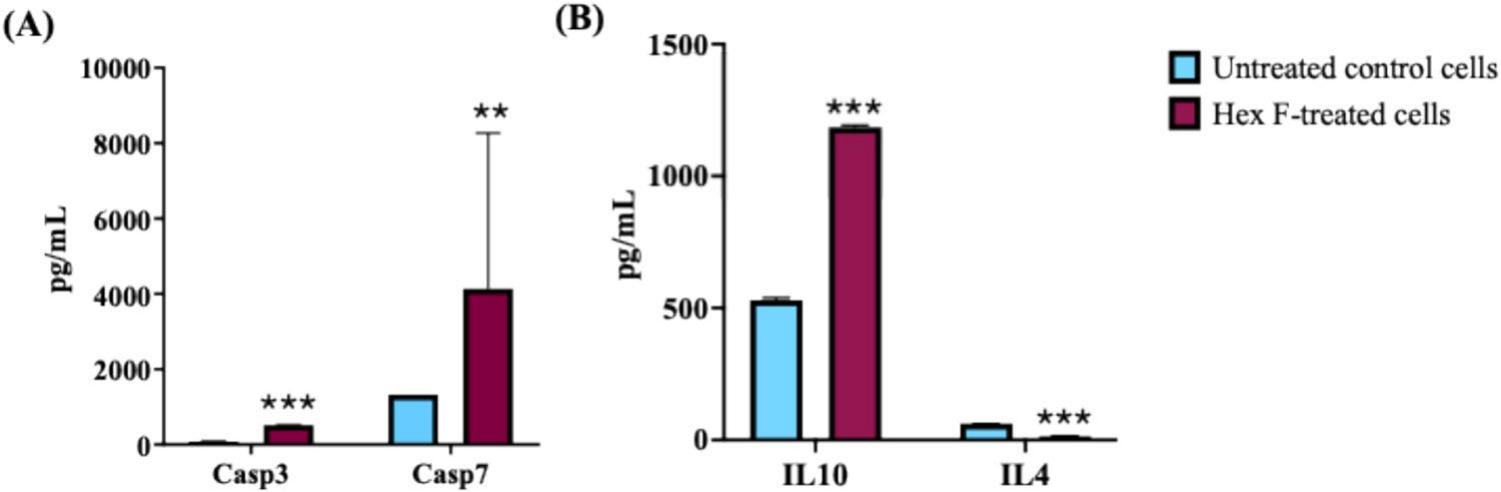
Protein expression analysis of caspases and inflammatory markers in HCT116 cells treated with *P. crispa* Hex F. Protein levels were quantified using specific ELISA kits according to manufacturer's protocols. Cells were treated with 39.4 μg/mL Hex F for 48 h. After treatment with *P. crispa* Hex F, cell lysates were prepared, and enzymatic activities were measured by a colorimetric assay for **A** Caspase activity analysis showing significant activation of executioner caspases: caspase-3 (Casp3) (primary executioner caspase responsible for DNA fragmentation and apoptotic body formation) and caspase-7 (Casp7) (secondary executioner caspase with similar function to caspase-3). **B** Inflammatory marker analysis demonstrating anti-inflammatory response: IL-10 (an anti-inflammatory cytokine that suppresses inflammatory responses) and IL-4 (pro-inflammatory cytokine involved in Th2 immune responses). Data represent mean ± SD of three independent experiments performed in triplicate. Statistical significance: **p < 0.005, and ***p < 0.001 compared to untreated control using independent t-test. Error bars represent standard deviation

Changes in the activities of caspases (Casp3 and Casp7) and anti-inflammatory markers IL-4 and IL-10 were assessed using colorimetric ELISA analysis in both treated and untreated HCT116 cells. The results revealed a significant increase in caspase-3 and caspase-7 levels in cells treated with *P. crispa* Hex F compared to untreated cells (Fig. 3A), indicating activation of apoptosis. On the other hand, the treatment with *P. crispa* Hex F significantly increased IL-10 levels while decreasing IL-4 levels, indicating a shift toward an anti-inflammatory response in the treated cells (Fig. 3B).

### Determination of oxidative stress markers

**Fig. 4:**
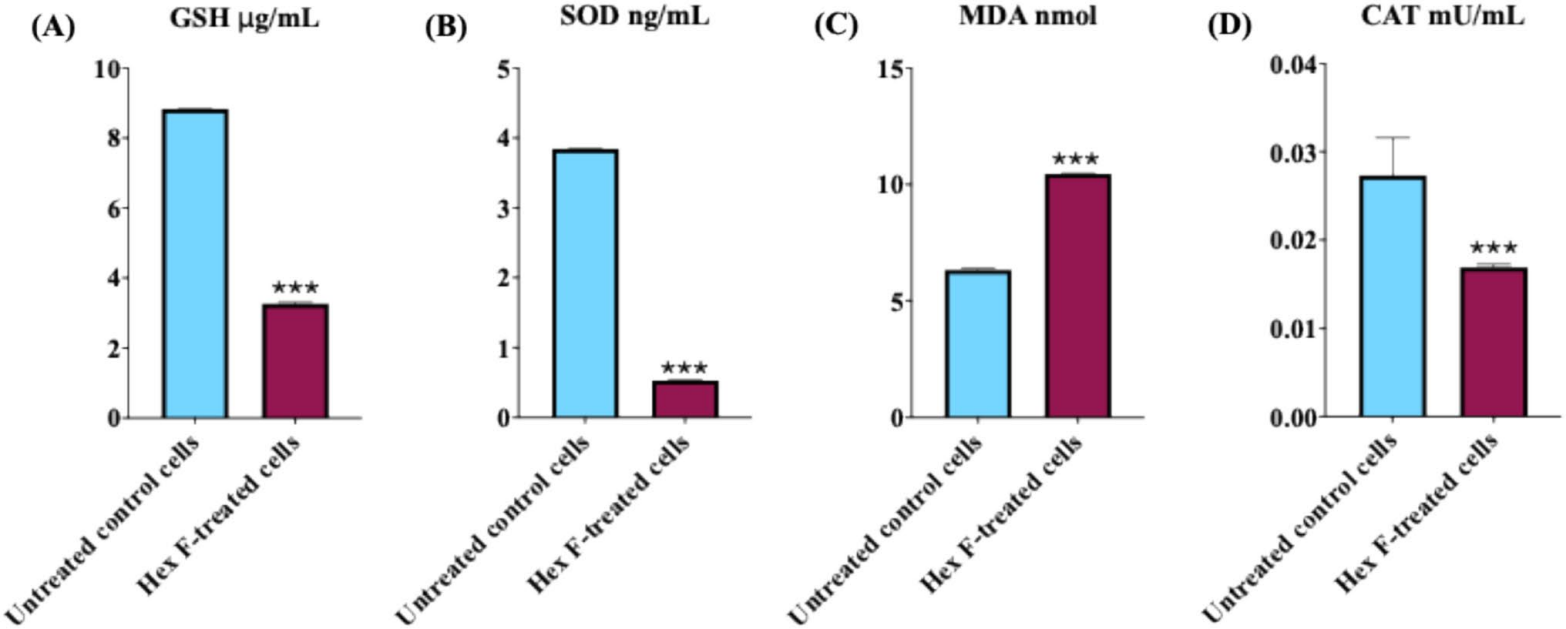
Oxidative stress marker analysis in HCT116 cells treated with *P. crispa* Hex F. Oxidative stress parameters were measured using specific colorimetric assay kits. Cells were treated with 39.4 μg/mL Hex F for 48 h. The antioxidant activities of **A** Reduced Glutathione (GSH) levels, a major intracellular antioxidant that protects cells from oxidative damage, **B** Superoxide Dismutase (SOD) activity, an enzyme that catalyzes the dismutation of superoxide radicals, **C** Malondialdehyde (MDA) levels, a biomarker of lipid peroxidation and oxidative damage, and **D** Catalase (CAT) activity, an enzyme that decomposes hydrogen peroxide. Data represent mean ± SD of three independent experiments performed in triplicate. Statistical significance: ***p < 0.001 compared to untreated control using independent t-test. Error bars represent standard deviation

The treatment with *P. crispa* Hex F significantly decreased the levels of GSH and SOD (Fig. 4), while MDA levels increased significantly, indicating oxidative stress in the HCT116 cells compared to untreated controls. However, CAT activity exhibited a non-significant decrease (Fig. 4).

### Determination of glycolytic pathway enzymes

**Fig. 5:**
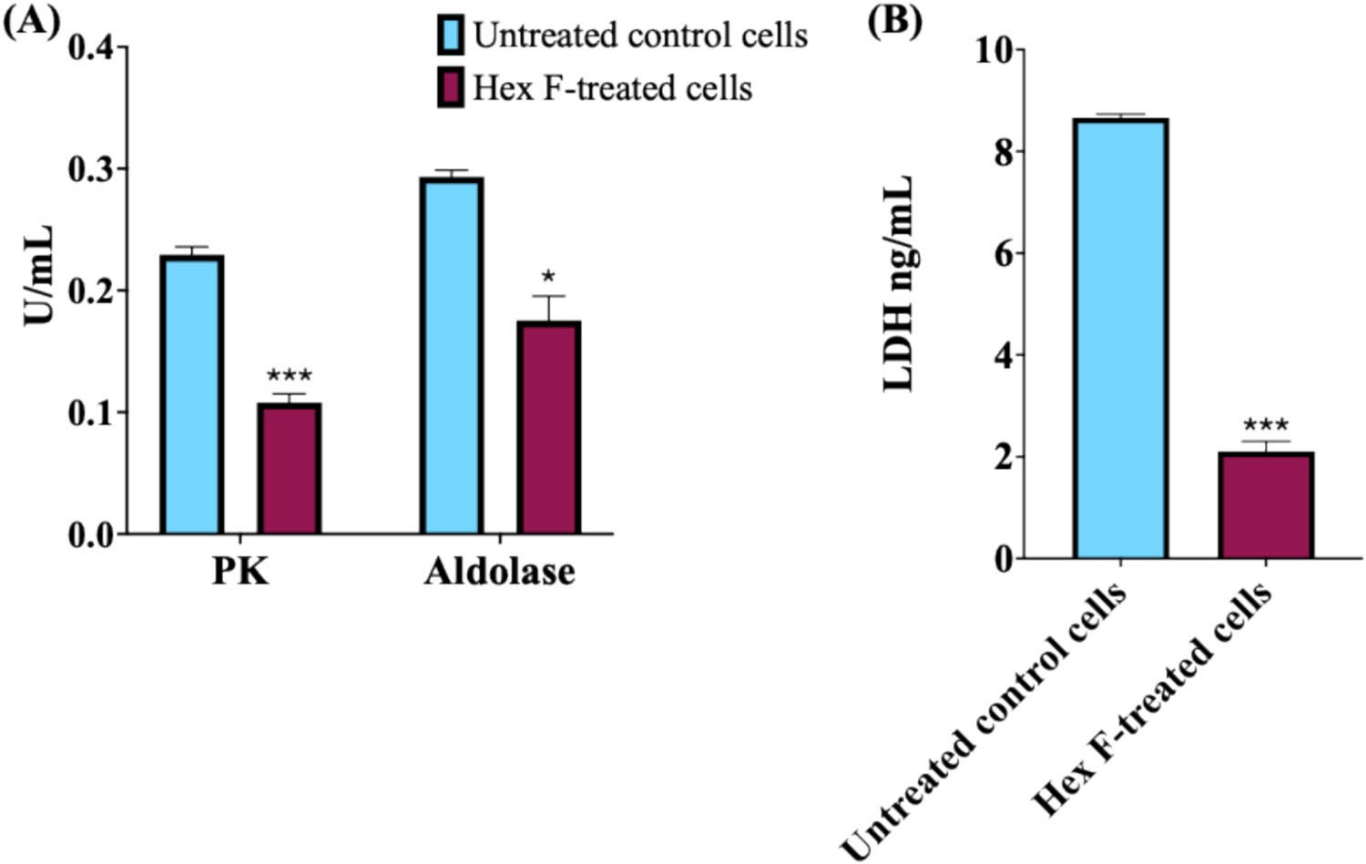
Glycolytic enzyme activity analysis in HCT116 cells treated with *P. crispa* Hex F. Key glycolytic enzymes were measured using specific activity assay kits to assess metabolic changes. Cells were treated with 39.4 μg/mL Hex F for 48 h. **A** Pyruvate Kinase (PK) activity, the rate-limiting enzyme that catalyzes the final step of glycolysis converting phosphoenolpyruvate to pyruvate, and Aldolase activity, an enzyme that cleaves fructose-1,6-bisphosphate into glyceraldehyde-3-phosphate and dihydroxyacetone phosphate, and **B** Lactate Dehydrogenase (LDH) activity, an enzyme that catalyzes the conversion of pyruvate to lactate in anaerobic conditions. The significant reduction in all three enzymes indicates substantial disruption of glycolytic metabolism and cellular energy production. Data represent mean ± SD of three independent experiments performed in triplicate. Statistical significance: *p < 0.05, and ***p < 0.001 compared to untreated control using independent t-test. Error bars represent standard deviation

The treatment of HCT116 cells with *P. crispa* Hex F significantly decreased the activities of pyruvate kinase (PK), aldolase, and lactate dehydrogenase (LDH), which are the key enzymes in the glycolytic pathway (Fig. 5). This indicates that Hex F reduced the glycolysis rate and impacted the cellular energy metabolism.

### Chemical profiling of *P. crispa* Hex F

As previously reported and confirmed through additional validation studies (Abo-Elghiet et al. 2023), GC–MS analysis of *P. crispa* Hex F identified 27 different compounds across various chemical classes, including hydrocarbons, sterols, alcohols, and fatty acids. The primary constituents identified were β-sitosterol (17.89%), phytol (15.65%), stigmasterol (13.13%), and lupeol (12.89%), which accounted for the highest concentrations. Compound identification was based on a comparison of mass spectral fragmentation patterns with those in the Wiley and NIST Mass Spectral Libraries.

## Discussion

The devastating impact of cancer, characterized by its complex biology and resistance to traditional treatments, underscores the urgent need for novel therapeutic options with recent estimates indicating that cancer will become the leading cause of death worldwide by 2030, surpassing cardiovascular diseases (Gao et al. 2024; Mahase 2019). This study evaluated the anti-proliferative potential of the Egyptian aerial parts of *P. crispa* MeOH Ex and its fractions against cancer cells. Among all fractions, Hex F and DCM F exhibited the most potent cytotoxic effects, with IC_50_ values ranging from 21.51 to 74.07 µg/mL against HepG2, HCT116, and Hep-2 cells. Notably, the *P. crispa* Hex F fraction exhibited a selectivity index (SI) of 1.7 against HCT116 cells, indicating a moderate but promising preference for cancer over normal cells. This suggests its potential as a lead candidate for chemo-protective or chemotherapeutic applications. These findings align with previous research highlighting the superior anticancer potential of non-polar fractions from medicinal plants, *P. crispa* essential oils, and extracts which typically contain lipophilic bioactive compounds with enhanced cellular penetration and target specificity (Kasote et al. 2024a; Mohamed Abdoul-Latif et al. 2023; Asma et al. 2022b). Consequently, the chemical profile and the possible anti-proliferative mechanisms of *P. crispa* Hex F were evaluated to further explore its therapeutic potential.

The chemical profile of *P. crispa* Hex F was determined using GC/MS, revealing a composition rich in sterols, with β-sitosterol, phytol, stigmasterol, and lupeol identified as the most abundant compounds (Abo-Elghiet et al. 2023). These compounds are known for their anticancer properties, including β-sitosterol reduces the risk of prostate, colon, and esophagus cancers by inducing apoptosis, arresting the cell cycle, and modulating oxidative stress (Wang et al. 2023). Phytol induces S-phase cell cycle arrest and increases ROS production (Islam et al. 2018), and stigmasterol promotes apoptosis and inhibits proliferation in various cancer cell lines (Zhang et al. 2022). Moreover, lupeol induces apoptosis and suppresses migration and invasion (Liu et al. 2021). The combined presence of these bioactive compounds in Hex F likely contributes to its observed cytotoxicity and anti-proliferative activity, supporting its potential as a therapeutic candidate. The observed selectivity index of 1.6 suggests that *P. crispa* Hex F demonstrates comparable selectivity to established anticancer agents. Recent studies have emphasized that natural products with SI values > 1.5 are considered promising candidates for further development, as they indicate preferential toxicity toward cancer cells while sparing normal tissues (Badisa et al. 2009). The significant induction of apoptosis observed in this study, with early apoptosis increased from 0.5267% to 7.8300%, and late apoptosis increased from 1.4% to 22.3%, demonstrates the potent pro-apoptotic effects of *P. crispa* Hex F. This level of apoptosis induction is comparable to that observed with established anticancer agents, such as paclitaxel and cisplatin, which typically induce 20–30% apoptosis in cancer cell lines at their IC_50_ concentrations. The dual induction of both early and late apoptosis suggests that the extract activates multiple apoptotic pathways, potentially involving both intrinsic (mitochondrial) and extrinsic (death receptor) pathways (Abed et al. 2021; Hanahan 2022; Hanahan and Weinberg 2011).

The cell cycle analysis revealed that *P. crispa* Hex F induces significant G_2_/M phase arrest in HCT116 cells while decreased in the G_0_/G_1_ phase which could be explained by the presence of phytol (Anoor et al. 2022), on the other hand, the number of cells in the S phase was decreased which may be due to the presence of stigmasterol (Wang et al. 2022). This finding is particularly significant as G_2_/M arrest is a critical checkpoint that prevents cells with damaged DNA from proceeding to mitosis, ultimately leading to apoptosis if the damage cannot be repaired. The G_2_/M checkpoint is regulated by key proteins including p53, ATM, and Chk1/Chk2 kinases, and disruption of this checkpoint is a hallmark of many anticancer agents. Recent research has shown that compounds inducing G_2_/M arrest often target microtubule dynamics, DNA repair mechanisms, or cell cycle regulatory proteins, making this a valuable therapeutic strategy (Matthews et al. 2022; Ciccia and Elledge 2010; Kastan and Bartek 2004).

Gene expression analysis provided crucial insights into the molecular mechanisms underlying the anticancer effects of *P. crispa* Hex F (Abed et al. 2021). The significant upregulation of p53 is particularly noteworthy as p53 is known as the "guardian of the genome" and plays a central role in DNA damage response, cell cycle arrest, and apoptosis induction with recent studies showing that p53 activation is essential for the efficacy of many natural anticancer compounds (Carneiro and El-Deiry 2020). The upregulation of both caspase-8 and caspase-9 indicates activation of both extrinsic and intrinsic apoptotic pathways, where β-sitosterol presence could be responsible for these upregulations along with other active compounds (Wang et al. 2023). These results suggest that *P. crispa* Hex F can trigger apoptosis through multiple mechanisms, which is advantageous for overcoming apoptosis resistance in cancer cells.

The downregulation of Bcl2 is significant which can be caused by the effect of lupeol (Prasad et al. 2018), as Bcl2 is a key anti-apoptotic protein that prevents mitochondrial membrane permeabilization and cytochrome c release with recent research demonstrating that Bcl2 downregulation is essential for effective cancer therapy and is associated with improved treatment outcomes (Singh et al. 2019). Lupeol presence was previously reported to affect IL4 (Vasconcelos et al. 2008), known to promote colorectal cancer progression (Koller et al. 2010), which underscores the extract's impact on apoptosis induction and tumor suppression. The simultaneous downregulation of CDK2, a pivotal regulator in this process (Bačević et al. 2017), and TopBP1, involved in P53 regulation (Liu et al. 2009), further supports the cell cycle arrest findings, knowing that these proteins are essential for S-phase progression and DNA replication, shedding light on additional mechanisms influenced by the Hexane fraction treatment, which may be due to the presence of stigmasterol (Zhang et al. 2022).

The protein-level analysis confirmed the gene expression findings, with significant increases in caspase-3 and caspase-7 activities. These executioner caspases are responsible for the final stages of apoptosis, including DNA fragmentation, protein cleavage, and apoptotic body formation (Julien and Wells 2017; McIlwain et al. 2015). The magnitude of caspase activation observed in this study is consistent with effective apoptosis induction and is comparable to levels achieved by clinically approved anticancer drugs. The inflammatory marker analysis revealed an interesting anti-inflammatory response, with IL-10 levels increasing, which may be due to the presence of lupeol (Abbas and Ali 2024) and IL-4 levels decreasing. This pattern suggests that *P. crispa* Hex F may help modulate the tumor microenvironment by promoting anti-inflammatory conditions (Greten and Grivennikov 2019), which can enhance immune system recognition and elimination of cancer cells. Recent studies have highlighted the importance of IL-10 in cancer therapy, as it can promote antitumor immunity while reducing inflammation-associated cancer progression (Greten and Grivennikov 2019; Saraiva et al. 2020).

The oxidative stress analysis provided evidence for another important mechanism of action. The significant decrease in GSH and SOD levels, coupled with increased MDA, indicates that *P. crispa* Hex F induces oxidative stress in cancer cells, which may be due to the presence of phytol compound for GSH (Lee et al. 2016), phytol for MDA (Islam et al. 2018). This is a well-established anticancer mechanism, as cancer cells are generally more susceptible to oxidative stress than normal cells due to their altered metabolism and reduced antioxidant capacity (Ding et al. 2021; Kuninaka et al. 2000). The selective induction of oxidative stress in cancer cells while sparing normal cells is a promising therapeutic strategy that has been successfully employed by several FDA-approved anticancer drugs (Perillo et al. 2020; Hayes et al. 2020).

PK is implicated in colorectal cancer treatment facilitation (Yin et al. 2020), aldolase is associated with metastasis (Bu et al. 2018), and high serum LDH levels are associated with a poor prognosis in many cancer types (Claps et al. 2022). In our study, the analysis revealed a significant disruption of cellular energy metabolism, with PK, Aldolase, and LDH activities significantly decreased because of the presence of both stigmasterol and β-sitosterol (Goswami et al. 2023). While CAT activity showed no significant change, where its role in antioxidant defense warrants further investigation. This finding is particularly relevant given the Warburg effect, where cancer cells rely heavily on glycolysis for energy production even in the presence of oxygen (Vander Heiden et al. 2009; Ganapathy-Kanniappan and Geschwind 2013). Targeting glycolytic metabolism has emerged as a promising anticancer strategy, as it can selectively affect cancer cells while having minimal impact on normal cells that can utilize oxidative phosphorylation. Recent clinical trials have shown that glycolytic inhibitors can enhance the efficacy of conventional chemotherapy and overcome drug resistance (Ganapathy-Kanniappan and Geschwind 2013; Liberti and Locasale 2016).

The chemical composition analysis identified β-sitosterol (17.89%), phytol (15.65%), stigmasterol (13.13%), and lupeol (12.89%) as the major bioactive compounds in *P. crispa* Hex F. These compounds have well-documented anticancer properties: β-sitosterol has been shown to induce apoptosis and inhibit tumor growth in various cancer models, stigmasterol exhibits anti-proliferative effects through cell cycle arrest, phytol demonstrates antioxidant and anti-inflammatory activities, and lupeol has been reported to inhibit cancer cell migration and invasion (Islam et al. 2018; Bin Sayeed and Ameen 2015; Gabay et al. 2010; Saleem 2009). The synergistic effects of these compounds likely contribute to the overall anticancer activity observed in this study, supporting the concept that whole plant extracts may be more effective than isolated compounds (Caesar and Cech 2019).

Comparative analysis with other *Pulicaria* species reveals that *P. crispa* demonstrates superior anticancer potential, with recent studies on *P. undulata* and *P. dysenterica* showing IC_50_ values ranging from 50–120 μg/mL against various cancer cell lines, indicating that *P. crispa* Hex F is among the most potent fractions within this genus (Venditti et al. 2015). The unique chemical profile of *P. crispa*, particularly its high content of sterols and terpenoids, may account for its enhanced bioactivity compared to other species (Al-Massarani et al. 2013).

From a translational perspective, the findings of this study provide a strong foundation for further development of *P. crispa* Hex F as an anticancer agent. The multiple mechanisms of action identified, including cell cycle arrest, apoptosis induction, oxidative stress generation, and metabolic disruption, suggest that this extract could be effective against various cancer types and may help overcome drug resistance mechanisms (Holohan et al. 2013). However, further studies are needed to evaluate the in vivo efficacy, pharmacokinetics, and safety profile of *P. crispa* Hex F before clinical translation (Atanasov et al. 2021).

The limitations of this study include the use of a single cancer cell line for mechanistic studies and the lack of in vivo validation (Begley and Ioannidis 2015). Future research should include multiple cancer cell lines, normal cell controls for all assays, and comprehensive animal studies to confirm the anticancer potential and safety of *P. crispa* Hex F. Additionally, structure–activity relationship studies of individual compounds and their combinations could provide insights for optimizing the therapeutic potential of this natural product (Newman and Cragg 2020).

## Conclusion

In conclusion, this study demonstrates that P. crispa Hex F exhibits significant anticancer activity against HCT116 colorectal cancer cells through multiple molecular mechanisms. The combination of cell cycle arrest, apoptosis induction, oxidative stress generation, and metabolic disruption makes this extract a promising candidate for natural anticancer drug development. The identification of specific bioactive compounds and their mechanisms of action provide a scientific basis for the traditional use of *P*. *crispa* and support its potential for modern cancer therapy (Thomford et al. 2018).

## Data Availability

The data that support the findings of this study, including raw experimental data, and gene expression data are available upon reasonable request. The data were used during the research but are not publicly available due to privacy and institutional policy restrictions. Any additional information regarding the study, including detailed protocols and statistical analysis upon reasonable request.

## References

[CR1] Abbas TF, Ali HZ (2024) Lupeol stimulates iNOS, TNF-α, and IL-10 expression in the U937 cell line infected with old-world Leishmania donovani. Cytokine. 10.1016/j.cyto.2024.156757 39288647 10.1016/j.cyto.2024.156757

[CR2] Abbott KL, Ali A, Casalena D, Do BT, Ferreira R, Cheah JH, Heiden MG, Vander. (2023) Screening in serum-derived medium reveals differential response to compounds targeting metabolism. Bio. 10.1101/2023.02.25.529972 10.1016/j.chembiol.2023.08.007 PMC10581593 37689063

[CR3] Abed N, El-hallouty S, El-Zayat E, El-Sherief A (2021) In vitro anticancer activity of nanazoxid drug against colorectal cancer cell line and its moclecular pathways. Egypt Arab J Appl Sci Technol. 10.21608/eajast.2021.89088.1000

[CR4] Abo-Elghiet F, Rushdi A, Ibrahim MH, Mahmoud SH, Rabeh MA, Alshehri SA, El Menofy NG (2023) Chemical profile, antibacterial, antibiofilm, and antiviral activities of pulicaria crispa most potent fraction: an in vitro and in silico study. Molecules 28(10):4184. 10.3390/molecules28104184 37241923 10.3390/molecules28104184 PMC10224259

[CR5] Aebi, H. (1984). Catalase in vitro (pp. 121–126). 10.1016/S0076-6879(84)05016-3

[CR6] Ali Abdalla YO, Subramaniam B, Nyamathulla S, Shamsuddin N, Arshad NM, Mun KS, Nagoor NH (2022) Natural products for cancer therapy: a review of their mechanism of actions and toxicity in the past decade. J Trop Med. 10.1155/2022/5794350 35309872 10.1155/2022/5794350 PMC8933079

[CR7] Al-Maqtari QA, Mahdi AA, Al-Ansi W, Mohammed JK, Wei M, Yao W (2021) Evaluation of bioactive compounds and antibacterial activity of Pulicaria jaubertii extract obtained by supercritical and conventional methods. J Food Meas Charact 15(1):449–456. 10.1007/s11694-020-00652-5

[CR8] Al-Massarani S, El Gamal A, Al-Musayeib N, Mothana R, Basudan O, Al-Rehaily A, Maes L (2013) Phytochemical antimicrobial and antiprotozoal evaluation of garcinia mangostana pericarp and α-mangostin, its major xanthone derivative. Molecules. 10.3390/molecules180910599 24002136 10.3390/molecules180910599 PMC6270423

[CR9] Alvarez-Sala A, Attanzio A, Tesoriere L, Garcia-Llatas G, Barberá R, Cilla A (2019) Apoptotic effect of a phytosterol-ingredient and its main phytosterol (β-sitosterol) in human cancer cell lines. Int J Food Sci Nutr 70(3):323–334. 10.1080/09637486.2018.1511689 30192685 10.1080/09637486.2018.1511689

[CR10] AlZain MN, Albarakaty FM, El-Desoukey RMA (2023) An ethnobotanical, phytochemical analysis, antimicrobial and biological studies of pulicaria crispa as a graze promising shrub. Life 13(11):2197. 10.3390/life13112197 38004337 10.3390/life13112197 PMC10672700

[CR11] Anand U, Dey A, Chandel AKS, Sanyal R, Mishra A, Pandey DK, Pérez de la Lastra JM (2023) Cancer chemotherapy and beyond: current status, drug candidates, associated risks and progress in targeted therapeutics. Gene Dis. 10.1016/j.gendis.2022.02.007 10.1016/j.gendis.2022.02.007 PMC10310991 37397557

[CR12] Anoor P, Yadav A, Rajkumar K, Kande R, Tripura C, Naik K, Burgula S (2022) Methanol extraction revealed anticancer compounds Quinic Acid, 2(5H)-Furanone and Phytol in *Andrographis paniculata*. Molecul Clin Oncol 17(5):151. 10.3892/mco.2022.2584 10.3892/mco.2022.2584 PMC9511679 36172002

[CR13] Asma ST, Acaroz U, Imre K, Morar A, Shah SRA, Hussain SZ, Ince S (2022a) Natural products/bioactive compounds as a source of anticancer drugs. Cancers. 10.3390/cancers14246203 36551687 10.3390/cancers14246203 PMC9777303

[CR14] Atanasov AG, Zotchev SB, Dirsch VM, Orhan IE, Banach M, Rollinger JM, Supuran CT (2021) Natural products in drug discovery: advances and opportunities. Nat Rev Drug Discov. 10.1038/s41573-020-00114-z 33510482 10.1038/s41573-020-00114-z PMC7841765

[CR15] Awad AB, Fink CS (2000) Phytosterols as anticancer dietary components: evidence and mechanism of action. J Nutr 130(9):2127–2130. 10.1093/jn/130.9.2127 10958802 10.1093/jn/130.9.2127

[CR16] Babushok VI, Linstrom PJ, Zenkevich IG (2011) Retention indices for frequently reported compounds of plant essential oils. J Phys Chem Refer Data. 10.1063/1.3653552

[CR17] Bačević K, Lossaint G, Achour TN, Georget V, Fisher D, Dulić V (2017) Cdk2 strengthens the intra-S checkpoint and counteracts cell cycle exit induced by DNA damage. Sci Rep. 10.1038/s41598-017-12868-5 29044141 10.1038/s41598-017-12868-5 PMC5647392

[CR18] Badisa RB, Darling-Reed SF, Joseph P, Cooperwood JS, Latinwo LM, Goodman CB (2009) Selective cytotoxic activities of two novel synthetic drugs on human breast carcinoma MCF-7 cells. Anticancer Res 29(8):2993–2996 19661306 PMC2885965

[CR19] Barabadi T, Rahimi H, Mohamadi-Zarch SM, Bagheri SM (2025) A comprehensive review on anticancer potential of *Pulicaria* plants and their derivatives. Curr Cancer Drug Target. 10.2174/0115680096358261250115114608 10.2174/0115680096358261250115114608 39945254

[CR20] Barnawi IO, Ali I (2019) Anticancer potential of pulicaria crispa extract on human breast cancer MDA-MB-231 cells. Lett Drug des Discovery 16(12):1354–1359. 10.2174/1570180816666190712110224

[CR21] Begley CG, Ioannidis JPA (2015) Reproducibility in science. Circ Res 116(1):116–126. 10.1161/CIRCRESAHA.114.303819 25552691 10.1161/CIRCRESAHA.114.303819

[CR22] Bergmeyer HU (1984) Enzymatische analyse neuer generation. Fresenius’ Zeitschrift Für Analytische Chemie 319(8):883–889. 10.1007/BF00487065

[CR23] Berridge MV, Herst PM, Tan AS (2005) Tetrazolium dyes as tools in cell biology: New insights into their cellular reduction. Biotechnol Annu Rev. 10.1016/S1387-2656(05)11004-7 16216776 10.1016/S1387-2656(05)11004-7

[CR24] Bhattacharjya R, Tyagi R, Dey S, Dutta A, Kumar A, El-Sheekh MM, Tiwari A (2025) In-vitro assessment and characterization of anticancer, antibacterial, and antioxidant activity of diatom-derived metabolites. Sci Rep. 10.1038/s41598-025-87472-z 40287447 10.1038/s41598-025-87472-z PMC12033239

[CR25] Bienvenu J, Monneret G, Fabien N, Revillard JP (2000) The clinical usefulness of the measurement of cytokines. CCLM. 10.1515/CCLM.2000.040 10928646 10.1515/CCLM.2000.040

[CR26] Bin Sayeed MS, Ameen SS (2015) Beta-sitosterol: a promising but orphan nutraceutical to fight against cancer. Nutr Cancer 67(8):1216–1222. 10.1080/01635581.2015.1087042 10.1080/01635581.2015.1087042 26473555

[CR27] Brevet A, Roustan C, Pradel L, van Thoai N (1975) Muscle pyruvate kinase: interaction with substrates and analogues studied by difference spectroscopy. Eur J Biochem 52(2):345–350. 10.1111/j.1432-1033.1975.tb04002.x 170088 10.1111/j.1432-1033.1975.tb04002.x

[CR28] Bu P, Chen K-Y, Xiang K, Johnson C, Crown SB, Rakhilin N, Shen X (2018) Aldolase B-mediated fructose metabolism drives metabolic reprogramming of colon cancer liver metastasis. Cell Metabol. 10.1016/j.cmet.2018.04.003 10.1016/j.cmet.2018.04.003 PMC5990465 29706565

[CR29] Bustin SA, Benes V, Garson JA, Hellemans J, Huggett J, Kubista M, Wittwer CT (2009) The MIQE guidelines: minimum information for publication of quantitative real-time pcr experiments. Clin Chemist. 10.1373/clinchem.2008.112797 10.1373/clinchem.2008.112797 19246619

[CR30] Caesar LK, Cech NB (2019) Synergy and antagonism in natural product extracts: when 1 + 1 does not equal 2. Nat Prod Rep 36(6):869–888. 10.1039/C9NP00011A 31187844 10.1039/c9np00011a PMC6820002

[CR31] Carneiro BA, El-Deiry WS (2020) Targeting apoptosis in cancer therapy. Nat Rev Clin Oncol 17(7):395–417. 10.1038/s41571-020-0341-y 32203277 10.1038/s41571-020-0341-y PMC8211386

[CR32] Chehelgerdi M, Chehelgerdi M, Allela OQB, Pecho RDC, Jayasankar N, Rao DP, Akhavan-Sigari R (2023) Progressing nanotechnology to improve targeted cancer treatment: overcoming hurdles in its clinical implementation. Mol Cancer. 10.1186/s12943-023-01865-0 37814270 10.1186/s12943-023-01865-0 PMC10561438

[CR33] Chomczynski P, Sacchi N (2006) The single-step method of RNA isolation by acid guanidinium thiocyanate–phenol–chloroform extraction: twenty-something years on. Nat Protoc 1(2):581–585. 10.1038/nprot.2006.83 17406285 10.1038/nprot.2006.83

[CR34] Chunarkar-Patil P, Kaleem M, Mishra R, Ray S, Ahmad A, Verma D, Kumar S (2024) Anticancer drug discovery based on natural products: from computational approaches to clinical studies. Biomedicines. 10.3390/biomedicines12010201 38255306 10.3390/biomedicines12010201 PMC10813144

[CR35] Ciccia A, Elledge SJ (2010) The DNA damage response: making it safe to play with knives. Mol Cell 40(2):179–204. 10.1016/j.molcel.2010.09.019 20965415 10.1016/j.molcel.2010.09.019 PMC2988877

[CR36] Claps G, Faouzi S, Quidville V, Chehade F, Shen S, Vagner S, Robert C (2022) The multiple roles of LDH in cancer. Nat Rev Clin Oncol 19(12):749–762. 10.1038/s41571-022-00686-2 36207413 10.1038/s41571-022-00686-2

[CR37] Cohen J (2013) Statistical power analysis for the behavioral sciences. Routledge. 10.4324/9780203771587

[CR38] Cossarizza A, Chang H, Radbruch A, Akdis M, Andrä I, Annunziato F, Zimmermann J (2017) Guidelines for the use of flow cytometry and cell sorting in immunological studies. Europ J Immunol. 10.1002/eji.201646632 10.1002/eji.201646632 PMC9165548 29023707

[CR39] Crowley LC, Marfell BJ, Scott AP, Waterhouse NJ (2016) Quantitation of apoptosis and necrosis by annexin v binding, propidium iodide uptake, and flow cytometry. Cold Spring Harb Protoc. 10.1101/pdb.prot087288 27803250 10.1101/pdb.prot087288

[CR40] de Jager W, Bourcier K, Rijkers GT, Prakken BJ, Seyfert-Margolis V (2009) Prerequisites for cytokine measurements in clinical trials with multiplex immunoassays. BMC Immunol 10(1):52. 10.1186/1471-2172-10-52 19785746 10.1186/1471-2172-10-52 PMC2761376

[CR41] Dekinash M, Beltagy A, El Naggar EMB, Khattab A, El Fiky F (2018) Chemical composition and biological activities of the essential oil of pulicaria crispa in the middle east. Delta Univer Scient J. 10.21608/dusj.2018.205304

[CR42] Ding Y, Dai Y, Wu M, Li L (2021) Glutathione-mediated nanomedicines for cancer diagnosis and therapy. Chem Eng J. 10.1016/j.cej.2021.12888034539224

[CR43] Dong R, Wang J, Guan R, Sun J, Jin P, Shen J (2025) Role of oxidative stress in the occurrence, development, and treatment of breast cancer. Antioxidants 14(1):104. 10.3390/antiox14010104 39857438 10.3390/antiox14010104 PMC11760893

[CR44] Dunn OJ (1961) Multiple comparisons among means. J Am Stat Assoc 56(293):52–64. 10.1080/01621459.1961.10482090

[CR45] Ellman GL (1959) Tissue sulfhydryl groups. Arch Biochem Biophys 82(1):70–77. 10.1016/0003-9861(59)90090-6 13650640 10.1016/0003-9861(59)90090-6

[CR46] Emran TB, Rahman MA, Uddin MMN, Rahman MM, Uddin MZ, Dash R, Layzu C (2015) Effects of organic extracts and their different fractions of five Bangladeshi plants on in vitro thrombolysis. BMC Complement Altern Med. 10.1186/s12906-015-0643-2 25902818 10.1186/s12906-015-0643-2 PMC4414290

[CR47] Eskander A, Blakaj DM, Dziegielewski PT (2018) Decision making in advanced larynx cancer: an evidenced based review. Oral Oncol 86:195–199. 10.1016/j.oraloncology.2018.09.019 30409301 10.1016/j.oraloncology.2018.09.019

[CR48] Evidente A (2024) Advances on anticancer fungal metabolites: sources, chemical and biological activities in the last decade (2012–2023). Natur Product Bioprospect 14(1):31. 10.1007/s13659-024-00452-0 10.1007/s13659-024-00452-0 PMC11093966 38743184

[CR49] Field, A. P. . (2012). *Discovering statistics using SPSS*. SAGE.

[CR50] Fleige S, Pfaffl MW (2006) RNA integrity and the effect on the real-time qRT-PCR performance. Mol Aspects Med 27(2–3):126–139. 10.1016/j.mam.2005.12.003 16469371 10.1016/j.mam.2005.12.003

[CR51] Foglia B, Turato C, Cannito S (2023) Hepatocellular carcinoma: latest research in pathogenesis detection and treatment. Intern J Mol Sci. 10.3390/ijms241512224 10.3390/ijms241512224 PMC10419038 37569600

[CR52] Freshney RI (2010) Culture of animal cells. Wiley. 10.1002/9780470649367

[CR53] Gabay O, Sanchez C, Salvat C, Chevy F, Breton M, Nourissat G, Berenbaum F (2010) Stigmasterol: a phytosterol with potential anti-osteoarthritic properties. Osteoarthrit Cartil. 10.1016/j.joca.2009.08.019 10.1016/j.joca.2009.08.019 19786147

[CR54] Ganapathy-Kanniappan S, Geschwind J-FH (2013) Tumor glycolysis as a target for cancer therapy: progress and prospects. Mol Cancer. 10.1186/1476-4598-12-152 24298908 10.1186/1476-4598-12-152 PMC4223729

[CR55] Gao H, Xi Z, Dai J, Xue J, Guan X, Zhao L, Xing F (2024) Drug resistance mechanisms and treatment strategies mediated by Ubiquitin-Specific Proteases (USPs) in cancers: new directions and therapeutic options. Mol Cancer. 10.1186/s12943-024-02005-y 38702734 10.1186/s12943-024-02005-y PMC11067278

[CR56] *Gas Chromatography and Mass Spectrometry*. (2011). Elsevier. 10.1016/C2009-0-17039-3

[CR57] Ghasemi M, Turnbull T, Sebastian S, Kempson I (2021) The MTT assay: utility, limitations, pitfalls, and interpretation in bulk and single-cell analysis. Int J Mol Sci. 10.3390/ijms222312827 34884632 10.3390/ijms222312827 PMC8657538

[CR58] Ghosh S, Solanki R, Bhatia D, Sankaranarayanan S (2025) Nanomaterials for delivery of medicinal plant extracts and phytochemicals: Potential applications and future perspectives. Plant Nano Biol 12:100161. 10.1016/j.plana.2025.100161

[CR59] Goswami M, Priya J, Gupta S, Das G, Verma SK (2023) A comprehensive update on phytochemistry, analytical aspects, medicinal attributes, specifications and stability of stigmasterol. Steroids. 10.1016/j.steroids.2023.109244 37137454 10.1016/j.steroids.2023.109244

[CR60] Greten FR, Grivennikov SI (2019) Inflammation and cancer: triggers, mechanisms, and consequences. Immunity 51(1):27–41. 10.1016/j.immuni.2019.06.025 31315034 10.1016/j.immuni.2019.06.025 PMC6831096

[CR61] Halliwell B, Gutteridge JMC (2015) Free radicals in biology and medicine. Oxford University Press. 10.1093/acprof:oso/9780198717478.001.0001

[CR62] Hanahan D (2022) Hallmarks of cancer: new dimensions. Cancer Discov 12(1):31–46. 10.1158/2159-8290.CD-21-1059 35022204 10.1158/2159-8290.CD-21-1059

[CR63] Hanahan D, Weinberg RA (2011) Hallmarks of cancer: the next generation. Cell 144(5):646–674. 10.1016/j.cell.2011.02.013 21376230 10.1016/j.cell.2011.02.013

[CR64] Harborne JB (1984) Phytochemical Methods. Springer, Netherlands, Dordrecht. 10.1007/978-94-009-5570-7

[CR65] Hayes JD, Dinkova-Kostova AT, Tew KD (2020) Oxidative stress in cancer. Cancer Cell 38(2):167–197. 10.1016/j.ccell.2020.06.001 32649885 10.1016/j.ccell.2020.06.001 PMC7439808

[CR66] Holohan C, Van Schaeybroeck S, Longley DB, Johnston PG (2013) Cancer drug resistance: an evolving paradigm. Nat Rev Cancer 13(10):714–726. 10.1038/nrc3599 24060863 10.1038/nrc3599

[CR67] Igissin N, Zatonskikh V, Telmanova Z, Tulebaev R, Moore M (2023) Laryngeal cancer: epidemiology, etiology, and prevention: a narrative review. Iran J Public Health. 10.18502/ijph.v52i11.14025 38106821 10.18502/ijph.v52i11.14025 PMC10719707

[CR68] Islam MT, Ali ES, Uddin SJ, Shaw S, Islam MA, Ahmed MI, Atanasov AG (2018) Phytol: a review of biomedical activities. Food Chem Toxicol. 10.1016/j.fct.2018.08.032 30130593 10.1016/j.fct.2018.08.032

[CR69] Jagtap GA, Badge A, Kohale MG, Wankhade RS (2023) The role of the biosafety cabinet in preventing infection in the clinical laboratory. Cureus. 10.7759/cureus.51309 38288229 10.7759/cureus.51309 PMC10823295

[CR70] Julien O, Wells JA (2017) Caspases and their substrates. Cell Death Differ 24(8):1380–1389. 10.1038/cdd.2017.44 28498362 10.1038/cdd.2017.44 PMC5520456

[CR71] Kasote DM, Nawaz MA, Usman K, Ullah N, Alsafran M (2024a) A critical review on Pulicaria species occurring in Qatar: traditional uses, phytochemistry and biological activities. Phytochem Rev. 10.1007/s11101-024-09932-0

[CR72] Kastan MB, Bartek J (2004) Cell-cycle checkpoints and cancer. Nature 432(7015):316–323. 10.1038/nature03097 15549093 10.1038/nature03097

[CR73] Kim KH, Sederstrom JM (2015) Assaying cell cycle status using flow cytometry. Curr Protoc Mol Biol. 10.1002/0471142727.mb2806s111 26131851 10.1002/0471142727.mb2806s111 PMC4516267

[CR74] Koch A, Tamez P, Pezzuto J, Soejarto D (2005) Evaluation of plants used for antimalarial treatment by the Maasai of Kenya. J Ethnopharmacol 101(1–3):95–99. 10.1016/j.jep.2005.03.011 15878245 10.1016/j.jep.2005.03.011

[CR75] Koller FL, Hwang DG, Dozier EA, Fingleton B (2010) Epithelial interleukin-4 receptor expression promotes colon tumor growth. Carcinogenesis 31(6):1010–1017. 10.1093/carcin/bgq044 20176658 10.1093/carcin/bgq044 PMC2878360

[CR76] Koopman G, Reutelingsperger CP, Kuijten GA, Keehnen RM, Pals ST, van Oers MH (1994) Annexin V for flow cytometric detection of phosphatidylserine expression on B cells undergoing apoptosis. Blood 84(5):1415–1420 8068938

[CR77] Kuninaka S, Ichinose Y, Koja K, Toh Y (2000) Suppression of manganese superoxide dismutase augments sensitivity to radiation, hyperthermia and doxorubicin in colon cancer cell lines by inducing apoptosis. Br J Cancer 83(7):928–934. 10.1054/bjoc.2000.1367 10970696 10.1054/bjoc.2000.1367 PMC2374675

[CR78] Kupchan SM, Tsou G, Sigel CW (1973) Datiscacin, a novel cytotoxic cucurbitacin 20-acetate from Datisca glomerata. J Org Chem 38(7):1420–1421. 10.1021/jo00947a041 4694234 10.1021/jo00947a041

[CR79] Laka K, Makgoo L, Mbita Z (2022) Cholesterol-lowering phytochemicals: targeting the mevalonate pathway for anticancer interventions. Front Genet. 10.3389/fgene.2022.841639 35391801 10.3389/fgene.2022.841639 PMC8981032

[CR80] Lakshmanan I, Batra S (2013) Protocol for apoptosis assay by flow cytometry using annexin V staining method. Bio-Protoc. 10.21769/BioProtoc.374 27430005 10.21769/bioprotoc.374 PMC4943750

[CR81] Larson EC, Pond CD, Rai PP, Matainaho TK, Piskaut P, Franklin MR, Barrows LR (2016) Traditional preparations and methanol extracts of medicinal plants from papua new guinea exhibit similar cytochrome P450 inhibition. Evid Based Compl Alternat Med. 10.1155/2016/7869710 10.1155/2016/7869710 PMC5013206 27642356

[CR82] Lee W, Woo E-R, Lee DG (2016) Phytol has antibacterial property by inducing oxidative stress response in *Pseudomonas aeruginosa*. Free Radical Res 50(12):1309–1318. 10.1080/10715762.2016.1241395 27667264 10.1080/10715762.2016.1241395

[CR83] Liberti MV, Locasale JW (2016) The warburg effect: how does it benefit cancer cells? Trends Biochem Sci 41(3):211–218. 10.1016/j.tibs.2015.12.001 26778478 10.1016/j.tibs.2015.12.001 PMC4783224

[CR84] Liu K, Bellam N, Lin H-Y, Wang B, Stockard CR, Grizzle WE, Lin W-C (2009) Regulation of p53 by TopBP1: a potential mechanism for p53 inactivation in cancer. Mol Cell Biol 29(10):2673–2693. 10.1128/MCB.01140-08 19289498 10.1128/MCB.01140-08 PMC2682038

[CR85] Liu L, Yang J, Shi Y (2010) ChemInform abstract: phytochemicals and biological activities of pulicaria species. ChemInform. 10.1002/chin.201021264 10.1002/cbdv.200900014 20151381

[CR86] Liu Z, Dang Z, Yin H, Liu Y (2021) Making waves: Improving removal performance of conventional wastewater treatment plants on endocrine disrupting compounds (EDCs): their conjugates matter. Water Res. 10.1016/j.watres.2020.116469 33011607 10.1016/j.watres.2020.116469

[CR87] Livak KJ, Schmittgen TD (2001) Analysis of relative gene expression data using real-time quantitative PCR and the 2−ΔΔCT method. Methods 25(4):402–408. 10.1006/meth.2001.1262 11846609 10.1006/meth.2001.1262

[CR88] Llovet JM, Kelley RK, Villanueva A, Singal AG, Pikarsky E, Roayaie S, Finn RS (2021) Hepatocellular carcinoma. Nat Rev Dis Primers. 10.1038/s41572-020-00240-3 33479224 10.1038/s41572-020-00240-3 PMC13537433

[CR89] Mahase E (2019) Cancer overtakes CVD to become leading cause of death in high income countries. BMJ. 10.1136/bmj.l5368 31481521 10.1136/bmj.l5368

[CR90] Mahmood MA, Abd AH, Kadhim EJ (2024) Assessing the cytotoxicity of phenolic and terpene fractions extracted from Iraqi Prunus arabica against AMJ13 and SK-GT-4 human cancer cell lines. Research. 10.12688/f1000research.131336.3 10.12688/f1000research.131336.3 PMC11480737 39416710

[CR91] Mansour GH, El-Magd MA, Mahfouz DH, Abdelhamid IA, Mohamed MF, Ibrahim NS, Elzayat EM (2021) Bee venom and its active component Melittin synergistically potentiate the anticancer effect of Sorafenib against HepG2 cells. Bioorgan Chem. 10.1016/j.bioorg.2021.105329 10.1016/j.bioorg.2021.105329 34544028

[CR92] Mardiana L, Milanda T, Hadisaputri YE, Chaerunisaa A (2025) Phytosome-enhanced secondary metabolites for improved anticancer efficacy: mechanisms and bioavailability review. Drug des Dev Ther 19:201–218. 10.2147/DDDT.S483404 10.2147/DDDT.S483404 PMC11734513 39816849

[CR93] Marley AR, Nan H (2016) Epidemiology of colorectal cancer. Intern J Mol Epidemiol Genet 7(3):105–114 PMC5069274 27766137

[CR94] Matthews HK, Bertoli C, de Bruin RAM (2022) Cell cycle control in cancer. Nat Rev Mol Cell Biol 23(1):74–88. 10.1038/s41580-021-00404-3 34508254 10.1038/s41580-021-00404-3

[CR95] McCord JM, Fridovich I (1969) Superoxide dismutase. An enzymic function for erythrocuprein (hemocuprein). J Biol Chem 244(22):6049–6055 5389100

[CR96] McIlwain DR, Berger T, Mak TW (2015) Caspase functions in cell death and disease: figure 1. Cold Spring Harb Perspect Biol. 10.1101/cshperspect.a026716 25833847 10.1101/cshperspect.a026716 PMC4382736

[CR97] van Meerloo J, Kaspers GJL, Cloos J (2011) Cell sensitivity assays: The MTT assay. Methods Mol Biol. 10.1007/978-1-61779-080-5_20 21516412 10.1007/978-1-61779-080-5_20

[CR98] Miller NJ, Rice-Evans C, Davies MJ, Gopinathan V, Milner A (1993) A novel method for measuring antioxidant capacity and its application to monitoring the antioxidant status in premature neonates. Clin Sci 84(4):407–412. 10.1042/cs0840407 10.1042/cs0840407 8482045

[CR99] Mohamed HRH, Michael M, Elberry Y, Magdy H, Ismail M, Eltayeb N, Diab A (2025) Induction of potent preferential cell death, severe DNA damage and p53-independent ROS-mediated mitochondrial apoptosis by CaTiO3NPs in HNO-97 tongue cancer cells. Naunyn-Schmiedeberg’s Arch Pharmacol. 10.1007/s00210-025-04323-4 10.1007/s00210-025-04323-4 PMC12678538 40464944

[CR100] Mohamed Abdoul-Latif F, Ainane A, Houmed Aboubaker I, Mohamed J, Ainane T (2023) Exploring the potent anticancer activity of essential oils and their bioactive compounds: mechanisms and prospects for future cancer therapy. Pharmaceuticals. 10.3390/ph16081086 37631000 10.3390/ph16081086 PMC10458506

[CR101] Mosmann T (1983) Rapid colorimetric assay for cellular growth and survival: Application to proliferation and cytotoxicity assays. J Immunol Methods 65(1–2):55–63. 10.1016/0022-1759(83)90303-4 6606682 10.1016/0022-1759(83)90303-4

[CR102] Nasr FA (2024) The potential cytotoxic and apoptotic effects of Pulicaria undulata (L.) C.A. Mey chloroform fraction on human lung adenocarcinoma A549 cells. Ann Phytomed InternJ. 10.54085/ap.2024.13.2.46

[CR103] Newman DJ, Cragg GM (2020) Natural products as sources of new drugs over the nearly four decades from 01/1981 to 09/2019. J Nat Prod 83(3):770–803. 10.1021/acs.jnatprod.9b01285 32162523 10.1021/acs.jnatprod.9b01285

[CR104] Nicholson DW, Ali A, Thornberry NA, Vaillancourt JP, Ding CK, Gallant M, Miller DK (1995) Identification and inhibition of the ICE/CED-3 protease necessary for mammalian apoptosis. Nature. 10.1038/376037a0 7596430 10.1038/376037a0

[CR105] Ohkawa H, Ohishi N, Yagi K (1979) Assay for lipid peroxides in animal tissues by thiobarbituric acid reaction. Anal Biochem 95(2):351–358. 10.1016/0003-2697(79)90738-3 36810 10.1016/0003-2697(79)90738-3

[CR106] Park GH, Park JH, Song HM, Eo HJ, Kim MK, Lee JW, Jeong JB (2014) Anti-cancer activity of Ginger (Zingiber officinale) leaf through the expression of activating transcription factor 3 in human colorectal cancer cells. BMC Complement Altern Med. 10.1186/1472-6882-14-408 25338635 10.1186/1472-6882-14-408 PMC4210498

[CR107] Perfetto SP, Chattopadhyay PK, Roederer M (2004) Seventeen-colour flow cytometry: unravelling the immune system. Nat Rev Immunol 4(8):648–655. 10.1038/nri1416 15286731 10.1038/nri1416

[CR108] Perillo B, Di Donato M, Pezone A, Di Zazzo E, Giovannelli P, Galasso G, Migliaccio A (2020) ROS in cancer therapy: the bright side of the moon. Exper Molecul Med. 10.1038/s12276-020-0384-2 10.1038/s12276-020-0384-2 PMC7062874 32060354

[CR109] Pomeranz Y, Meloan CE (1994) Enzymatic Methods. Food Analysis. Springer, US, Boston, MA, pp 506–531

[CR110] Prasad N, Sabarwal A, Yadav UCS, Singh RP (2018) Lupeol induces S-phase arrest and mitochondria-mediated apoptosis in cervical cancer cells. J Biosci 43(2):249–261. 10.1007/s12038-018-9743-8 29872014

[CR111] Prommaban A, Utama-ang N, Chaikitwattana A, Uthaipibull C, Porter JB, Srichairatanakool S (2020) Phytosterol, lipid and phenolic composition, and biological activities of guava seed oil. Molecules 25(11):2474. 10.3390/molecules25112474 32471050 10.3390/molecules25112474 PMC7321134

[CR112] Raafat SN, El Wahed SA, Badawi NM, Saber MM, Abdollah MRA (2023) Enhancing the anticancer potential of metformin: fabrication of efficient nanospanlastics, in vitro cytotoxic studies on HEP-2 cells and reactome enhanced pathway analysis. Int J Pharm. 10.1016/j.ijpx.2023.100215 10.1016/j.ijpx.2023.100215 PMC10630776 38024451

[CR113] Rao, MRP, Ghadge I, Kulkarni SR Madgulkar, A. (2025). Importance of plant secondary metabolites in modern therapy (pp. 25–55). 10.1007/978-3-031-51158-5_5

[CR114] Razgonova MP, Zakharenko AM, Gordeeva EI, Shoeva OYu, Antonova EV, Pikula KS, Golokhvast KS (2021) Phytochemical analysis of phenolics, sterols, and terpenes in colored wheat grains by liquid chromatography with tandem mass spectrometry. Molecules. 10.3390/molecules26185580 34577050 10.3390/molecules26185580 PMC8469967

[CR115] Ririe KM, Rasmussen RP, Wittwer CT (1997) Product differentiation by analysis of DNA melting curves during the polymerase chain reaction. Anal Biochem 245(2):154–160. 10.1006/abio.1996.9916 9056205 10.1006/abio.1996.9916

[CR116] Roukos V, Pegoraro G, Voss TC, Misteli T (2015) Cell cycle staging of individual cells by fluorescence microscopy. Nat Protoc 10(2):334–348. 10.1038/nprot.2015.016 25633629 10.1038/nprot.2015.016 PMC6318798

[CR117] Saleem M (2009) Lupeol, a novel anti-inflammatory and anti-cancer dietary triterpene. Cancer Lett 285(2):109–115. 10.1016/j.canlet.2009.04.033 19464787 10.1016/j.canlet.2009.04.033 PMC2764818

[CR118] Saraiva M, Vieira P, O’Garra A (2020) Biology and therapeutic potential of interleukin-10. J Exp Med. 10.1084/jem.20190418 31611251 10.1084/jem.20190418 PMC7037253

[CR119] Sebaugh JL (2011) Guidelines for accurate EC50/IC50 estimation. Pharm Stat 10(2):128–134. 10.1002/pst.426 22328315 10.1002/pst.426

[CR120] Seibert E, & Tracy T.S. (2014). Fundamentals of Enzyme Kinetics (pp. 9–22). 10.1007/978-1-62703-758-7_2 10.1007/978-1-62703-758-7_2 24523106

[CR121] Sharifi-Rad J, Ozleyen A, Boyunegmez Tumer T, Oluwaseun Adetunji C, El Omari N, Balahbib A, Cho C (2019) Natural products and synthetic analogs as a source of antitumor drugs. Biomolecules. 10.3390/biom9110679 31683894 10.3390/biom9110679 PMC6920853

[CR122] Shukla S, Shukla AK, Upadhyay AM, Ray N, Fahad FI, Nagappan A, Mongre RK (2025) Molecular insight and antioxidative therapeutic potentials of plant-derived compounds in breast cancer treatment. Onco. 10.3390/onco5020027

[CR123] Siegel RL, Miller KD, Jemal A (2020) Cancer statistics, 2020. CA Cancer J Clin. 10.3322/caac.2159032940362

[CR124] Singh R, Letai A, Sarosiek K (2019) Regulation of apoptosis in health and disease: the balancing act of BCL-2 family proteins. Nat Rev Mol Cell Biol 20(3):175–193. 10.1038/s41580-018-0089-8 30655609 10.1038/s41580-018-0089-8 PMC7325303

[CR125] Sparkman OD (1997) Identification of essential oil components by gas chromatography / mass spectroscopy Robert P. Adams. J Am Soci Mass Spectro. 10.1016/S1044-0305(97)00026-3

[CR126] Stockert JC, Blázquez-Castro A, Cañete M, Horobin RW, Villanueva Á (2012) MTT assay for cell viability: intracellular localization of the formazan product is in lipid droplets. Acta Histochem 114(8):785–796. 10.1016/j.acthis.2012.01.006 22341561 10.1016/j.acthis.2012.01.006

[CR127] Talib WH, Alsayed AR, Barakat M, Abu-Taha MI, Mahmod AI (2021) Targeting drug chemo-resistance in cancer using natural products. Biomedicines 9(10):1353. 10.3390/biomedicines9101353 34680470 10.3390/biomedicines9101353 PMC8533186

[CR128] Thomford NE, Senthebane DA, Rowe A, Munro D, Seele P, Maroyi A, Dzobo K (2018) Natural products for drug discovery in the 21st century: innovations for novel drug discovery. Int J Mol Sci. 10.3390/ijms19061578 29799486 10.3390/ijms19061578 PMC6032166

[CR129] Vander Heiden MG, Cantley LC, Thompson CB (2009) Understanding the warburg effect: the metabolic requirements of cell proliferation. Science 324(5930):1029–1033. 10.1126/science.1160809 19460998 10.1126/science.1160809 PMC2849637

[CR130] Vaou N, Stavropoulou E, Voidarou C, Tsakris Z, Rozos G, Tsigalou C, Bezirtzoglou E (2022) Interactions between medical plant-derived bioactive compounds: focus on antimicrobial combination effects. Antibiotics. 10.3390/antibiotics11081014 36009883 10.3390/antibiotics11081014 PMC9404952

[CR131] Vasconcelos JF, Teixeira MM, Barbosa-Filho JM, Lúcio ASSC, Almeida JRGS, de Queiroz LP, Soares MBP (2008) The triterpenoid lupeol attenuates allergic airway inflammation in a murine model. Intern Immunopharmacol. 10.1016/j.intimp.2008.04.011 10.1016/j.intimp.2008.04.011 18602067

[CR132] Venditti A, Bianco A, Quassinti L, Bramucci M, Lupidi G, Damiano S, Maggi F (2015) Phytochemical analysis biological activity and secretory structures of stachys annua (L.) L subsp annua (Lamiaceae) from Central Italy. Chemist Biodivers. 10.1002/cbdv.201400275 10.1002/cbdv.201400275 26265569

[CR133] Vermeulen J, De Preter K, Lefever S, Nuytens J, De Vloed F, Derveaux S, Vandesompele J (2011) Measurable impact of RNA quality on gene expression results from quantitative PCR. Nucl Acid Res. 10.1093/nar/gkr065 10.1093/nar/gkr065 PMC3089491 21317187

[CR134] Wang W-L, Chen S-M, Lee Y-C, Chang W-W (2022) Stigmasterol inhibits cancer stem cell activity in endometrial cancer by repressing IGF1R/mTOR/AKT pathway. J Funct Foods. 10.1016/j.jff.2022.105338

[CR135] Wang H, Wang Z, Zhang Z, Liu J, Hong L (2023) β-Sitosterol as a promising anticancer agent for chemoprevention and chemotherapy: mechanisms of action and future prospects. Adv Nutr 14(5):1085–1110. 10.1016/j.advnut.2023.05.013 37247842 10.1016/j.advnut.2023.05.013 PMC10509430

[CR136] Wroblewski F, Ladue JS (1955) Lactic dehydrogenase activity in blood. Exp Biol Med 90(1):210–213. 10.3181/00379727-90-21985 10.3181/00379727-90-21985 13273400

[CR137] Yang JD, Hainaut P, Gores GJ, Amadou A, Plymoth A, Roberts LR (2019) A global view of hepatocellular carcinoma: trends, risk, prevention and management. Nat Rev Gastroenterol Hepatol 16(10):589–604. 10.1038/s41575-019-0186-y 31439937 10.1038/s41575-019-0186-y PMC6813818

[CR138] Yin C, Lu W, Ma M, Yang Q, He W, Hu Y, Xia L (2020) Efficacy and mechanism of combination of oxaliplatin with PKM2 knockdown in colorectal cancer. Oncol Lett 20(6):1–1. 10.3892/ol.2020.12175 33093921 10.3892/ol.2020.12175 PMC7573921

[CR139] Zafar A, Khatoon S, Khan MJ, Abu J, Naeem A (2025) Advancements and limitations in traditional anti-cancer therapies: a comprehensive review of surgery, chemotherapy, radiation therapy, and hormonal therapy. Discover Oncol 16(1):607. 10.1007/s12672-025-02198-8 10.1007/s12672-025-02198-8 PMC12021777 40272602

[CR140] Zhang X, Wang J, Zhu L, Wang X, Meng F, Xia L, Zhang H (2022) Advances in Stigmasterol on its anti-tumor effect and mechanism of action. Front Oncol. 10.3389/fonc.2022.1101289 36578938 10.3389/fonc.2022.1101289 PMC9791061

